# Estrous Cycle Dependent Fluctuations of Regulatory Neuropeptides in the Lower Urinary Tract of Female Rats upon Colon-Bladder Cross-Sensitization

**DOI:** 10.1371/journal.pone.0094872

**Published:** 2014-05-01

**Authors:** Xiao-Qing Pan, Anna P. Malykhina

**Affiliations:** Division of Urology, Department of Surgery, University of Pennsylvania, Glenolden, Pennsylvania, United States of America; Louisiana State University Health Sciences center, United States of America

## Abstract

Co-morbidity of bladder, bowel, and non-specific pelvic pain symptoms is highly prevalent in women. Little evidence is present on modulation of pelvic pain syndromes by sex hormones, therefore, the objective of this study was to clarify the effects of hormonal fluctuations within the estrous cycle on regulatory neuropeptides in female rats using a model of neurogenic bladder dysfunction. The estrous cycle in female rats (Sprague-Dawley, 230–250 g) was assessed by vaginal smears and weight of uterine horns. Neurogenic bladder dysfunction was induced by a single inflammatory insult to the distal colon. Protein expression of calcitonin gene related peptide (CGRP), substance P (SP), nerve growth factor (NGF), and brain derived neurotrophic factor (BDNF) in the pelvic organs, sensory ganglia and lumbosacral spinal cord was compared in rats in proestrus (high estrogen) *vs* diestrus (low estrogen). Under normal physiological conditions, concentration of SP and CGRP was similar in the distal colon and urinary bladder during all phases of the estrous cycle, however, acute colitis induced a significant up-regulation of CGRP content in the colon (by 63%) and urinary bladder (by 54%, p≤0.05 to control) of rats in proestrus. These changes were accompanied by a significant diminution of CGRP content in L6-S2 DRG after colonic treatment, likely associated with its release in the periphery. In rats with high estrogen at the time of testing (proestrus), experimental colitis caused a significant up-regulation of BDNF colonic content from 26.1±8.5 pg/ml to 83.4±32.5 pg/ml (N = 7, p≤0.05 to control) and also induced similar effects on BDNF in the urinary bladder which was also up-regulated by 5-fold in rats in proestrus (p≤0.05 to respective control). Our results demonstrate estrous cycle dependent fluctuations of regulatory neuropeptides in the lower urinary tract upon colon-bladder cross-sensitization, which may contribute to pain fluctuations in female patients with neurogenic bladder pain.

## Introduction

Chronic pelvic pain (CPP) affects up to 2.5 million women [1] and is more common in women than migraine or asthma [2], [3]. One third of women with CPP are diagnosed with bladder pain syndrome (BPS) [1] and 85% of women with CPP complain of pain of bladder origin [4]. Recent clinical and translational studies provide evidence that co-occurrence of bladder, bowel and non-specific pelvic pain symptoms may be due to development of cross-sensitization in the pelvis via neural mechanisms [5]–[7] and on the possible modulation of such pain by sex hormones [8]. Our previous animal data from males established an association between inflammation in the pelvis, release of pro-inflammatory neuropeptides from pelvic afferents, and development of pelvic organ cross-sensitization [9], [10].

Regulatory neuropeptides serve as the modulatory factors which contribute to visceral hyperalgesia and pain transmission resulting from noxious stimulation of the pelvic organs. The main neuropeptides contributing to pelvic pain upon pathological conditions include Substance P (SP), calcitonin gene-related peptide (CGRP), nerve growth factor (NGF), and brain-derived neurotrophic factor (BDNF) [11], [12]. Release of SP and CGRP from afferent terminals leads to the development of neurogenic inflammation in the affected viscera associated with degranulated mast cells, local plasma extravasation and arteriolar vasodilation [13]. We previously established that SP and CGRP contribute to long-lasting sensitization of neural pathways innervating the lower urinary tract (LUT) after transient inflammatory insult to the distal colon, thereby, causing neurogenic bladder dysfunction [9].

Increased visceral sensitivity after peripheral inflammation insult also correlates with an up-regulation of neurotrophins NGF [14]–[16] and BDNF [17]–[19] in the directly affected pelvic organs. Increased NGF content was detected in the urothelium of biopsies obtained from women with bladder pain syndrome (BPS) [15] while increased NGF, neurotrophin-3, and glial cell line–derived neurotrophic factor were found in the urine of BPS patients [20]. Recent animal studies with systemic drug intervention by injecting antiserum of either NGF or BDNF to rats with colitis suggested a possible interaction of the NGF and BDNF pathways in colitis-induced visceral hypersensitivity [21]. Additionally, BDNF generated within sensory ganglia can undergo anterograde transport to the central nerve terminals in the spinal dorsal horn where its release can increase synapse efficacy thereby contributing to central sensitization [22].

Steroidal hormones have the capacity to modulate neurogenic inflammation through interactions with a variety of pain and inflammation related mediators. Estradiol can regulate the expression of several neuropeptides including preprotachykinin [23], CGRP [24], and NGF [25] in sensory ganglia innervating the pelvic viscera. Functional studies established that under normal physiological conditions the effects of ovarian hormones on the function of different pelvic structures seem to have a minimal impact, but become more prominent upon pathological changes in one of the pelvic organs. For instance, no estrous influences on the micturition threshold in uninflamed bladder were previously determined in female rats, however, bladder inflammation substantially lowered the micturition threshold in proestrus and estrus [26]. Experiments with extravasation of Evans Blue dye revealed increased vascular permeability in the intact urinary bladder in proestrus after either colonic or uterine inflammation, however, these changes were insignificant in rats during metestrus [27]. In the present study, we aimed to further clarify the relationship between ovarian hormones, neurogenic inflammation in the pelvis and regulatory neuropeptides SP, CGRP, NGF and BDNF in female rats using a model of neurogenic bladder dysfunction induced by pelvic organ cross-sensitization.

## Materials and Methods

### Animals and Experimental Groups

Adult female Sprague–Dawley rats (230–250 g, Charles River Laboratories, Malvern, PA, 9–10 weeks of age) were maintained on a 12-h light/dark cycle and housed individually with *ad libitum* access to food and water. All protocols were approved by the University of Pennsylvania Institutional Animal Care and Use Committee and adhered to the guidelines for experimental pain in animals published by the International Association for the Study of Pain. Animals were divided into two experimental groups: 1- control group; and 2– transient colonic inflammation (experimental colitis) induced by TNBS (2,4,6-trinitrobenzene sulfonic acid). Control group of rats received saline and colitis group included TNBS containing enema (colon). Animals were sacrificed at 5 days after the treatment. Tissue samples from the distal colon, urinary bladder (detrusor), lumbosacral L6-S2 spinal cord and L6-S2 dorsal root ganglia (DRG) were collected from control and treated female rats in either diestrus (low estrogen) or proestrus (high estrogen) phases of the rat cycle. The protein concentrations of CGRP, SP, BDNF, and NGF were measured using commercially available ELISA kits.

### Estrous Cycle Phases

The stage of the estrous cycle for each female rat was determined by histological examination of cells in vaginal smears taken daily at 10 a.m. to 11∶00 am. It is well established [28] that the rat estrous cycle consists of four stages: diestrus 1 (D1, also referred to as metestrus), diestrus 2 (D2), proestrus (P), and estrus (E). The phase of the estrous cycle was determined based on a cytological profile of vaginal smears as described by Becker et al. [28]. Diestrus 1 (metestrus) was characterized by the lack of cells except for a few leukocytes; diestrus 2 (diestrus) phase included mainly leukocytes with few larger round cells, proestrus was presented by nucleated epithelial cells, and estrus was evident from the abundance of cornified cells. Using this technique, we were able to classify the stages of the estrous cycle in each rat after 3–4 cycles of observation. Group assignments were based on the phase of the estrous cycle the animal was in at the time of testing based on histological evaluation of vaginal smears. Comparisons between the proestrus and diestrus stages were made to give more explicit results [29], [30]. Rats in estrus were not included in the analysis due to potential cofounding effects of high estrogen in proestrus and its prolonged effects on many physiological functions as previously outlined [29]–[31].

### TNBS Model of Experimental Colitis

Transient colonic inflammation was induced by a single intracolonic administration of TNBS solution. The TNBS solution was prepared fresh before the instillation procedure. For 1 ml of the final solution 0.25 ml of TNBS (5% w/v, Sigma) 0.25 ml of water and 0.5 ml of ethanol (C_2_H_5_OH, Sigma) were mixed. The final concentration of TNBS was 12.5 mg/ml. Rats were fasted for 24 hours before instillation procedure to provide better access to the colonic lumen. Animals were briefly anesthetized with isoflurane (VEDCO Inc., St.Joseph, MO), a 7–8 cm long catheter made of polyethylene tubing and attached to a 1 cc syringe was inserted into the rat colon for enema administration. After instillation procedure, an animal was held by the tail to avoid any spill of instilled liquid. To assess the severity of developed inflammation, the daily Disease Activity Index (DAI) was calculated followed by MPO assay as previously described [32].

### Protein Extraction from the Tissues

Frozen tissues from control and treated rats were homogenized using PowerGen 500 homogenizer (Fisher Scientific, Rockford, IL) in ice-cold lysis buffer containing 25% glycerol, 62.5 mMTris-HCl, 1xprotease inhibitors (Roche, Complete mini) and phosphotase inhibitors (Roche, PhosSTOP). 10% SDS was added to the samples, vortexed and boiled for 4 min. The extracts were centrifuged at 10,000 rpm for 15 min at 4°C, and supernatants with the total protein were collected. Protein concentration in each sample was detected using BCA protein assay kit (Thermo Fisher Scientific, Rockford, IL). Bovine serum albumin (BSA) was used to generate the standard curve. Each protein sample was diluted 1∶20 with 1% SDS. All standards and samples were run in duplicate. The absorbance was measured at 562 nm on the Synergy 2 Multi-Detection Microplate Reader (BioTek Instruments, Winooski, VT) and data analysis was performed using Gen5 Microplate Data Collection & Analysis Software (BioTek Instruments, Winooski, VT).

### MPO Assay

To assess the severity of the inflammatory reaction in the pelvic organs, we ran the MPO assay. It is a widely used method of quantification of the level of developed inflammation based on the assessment of an enzyme found in neutrophils which relocate to the site of inflammation [33]. Urinary bladder and colonic tissue samples were homogenized in 2 ml of phosphate buffer (PB, pH = 6.0, 50 mM) with HTAB (hexadecyltrimethylammonium bromide, 0.5% Sigma, St.Louis). 1 ml of each homogenate was transferred to eppendorf tubes and underwent 3 cycles of freeze-thawing followed by sonication for 10 s. After 15 min of centrifugation at 12000×g (4°C), supernatant was collected and used to determine the total protein concentrations for all control and experimental groups. The total protein used for the assay was 200 µg/ml. The assay was started in a 96-well microplate using human MPO (Alpeco, Salem, NH) as a standard, 25 µl of total protein from each sample (200 µg/ml, colon and urinary bladder) and 25 µl of 3,3′-5,5′–tetramethylbenzidine (TMB; dissolved in DMSO, 1.6 mM), and incubated at 37°C for 5 min. 100 µL H_2_O_2_, dissolved in PB (0.05 M Na_3_PO_4_, 0.5% HETAB, pH 5.4) in a final concentration of 0.003% v/v was added, and the plate was incubated at 37°C for 5 min. The reaction was stopped by adding 100 µL of H_2_SO_4_ (4 M). The optical density value of each sample was read at 450 nm on a Multiscan EX spectrophotometer (Thermo Fisher Scientific, Waltham, MA) and converted into MPO values by using curves obtained from a standard sample of human MPO. Fold increase in MPO activity corresponded to the level of developed inflammatory reaction.

### Neuropeptide ELISAs

The levels of SP in harvested tissues were measured using a rat Substance P ELISA kit (MD Biosciences Inc., St. Paul, MN) according to the manufacturer’s instructions. The concentration of the total protein for SP assay was 200 µg/ml. Briefly, a 96-well microplate was loaded with 25 µl of primary antibody specific for rat SP. 50 µl of each sample and 50 µl of the standard SP dilutions (as a control) mixed in the assigned wells in duplicate followed by the addition of biotinylated SP into each well except the Blank. The plate was incubated for 2 h at RT, and then washed 6 times with provided in the kit wash buffer. Subsequently, 100 µl of biotinylated anti-SP antibody solution was added, incubated for 1 h, and then washed four times. 100µl of streptavidin-horseradish peroxidase conjugate solution was added to each well except chromogen blank, incubated for 1 hour, and washed again. After that, 100 µl of substrate solution provided in the kit was added to each well and the plate was incubated for 1 hour at room temperature. The reaction was stopped with 2N HCl and the optical density values were read at 450 nm using a Biotek Synergy 2 plate reader (BioTek Instruments Inc., Winooski, VT).

Tissue CGRP levels were measured using CGRP Enzyme Immunoassay (EIA) kit for rats (ALPCO Diagnostic, Salem, NH) according to the manufacturer’s instructions. For CGRP assay the total protein concentration was 400 µg/ml for the pelvic organs, and 300 µg/ml for the spinal cord and DRG. BDNF ELISA kit from Leinco Technologies (St. Louis, Missouri) was used to measure the concentration of BDNF, and NGF Emax Immunoassay System kit for rat was used for NGF detection (Cat.#TB226, Promega Corporation, Madison, WI). The concentration of the total protein for NGF and BDNF assays was 300 µg/ml.

### Chemicals

TNBS was purchased from Sigma Aldrich (St. Louis, MO). SP ELISA kit was purchased from MD Biosciences (St. Paul, MN), CGRP ELISA kit from ALPCO Diagnostics (Salem, NH), BDNF ELISA kit from Leinco Technologies (St. Louis, MO), and rat NGF Emax Immunoassay System kit from Promega Corporation (Madison, WI). BCA kit was obtained from Thermo Fisher Scientific (Waltham, MA).

### Statistical Analyses

All data are expressed as the mean±standard error of the mean (S.E.M). The protein concentrations of neuropeptides were statistically analyzed using one-way repeated measures ANOVA followed by group comparisons using Bonferroni’s post-hoc analysis (Systat Software Inc.,San Jose,CA).

## Results

### Phase of the Estrous Cycle and Effects of Colitis

**Figure 1:**
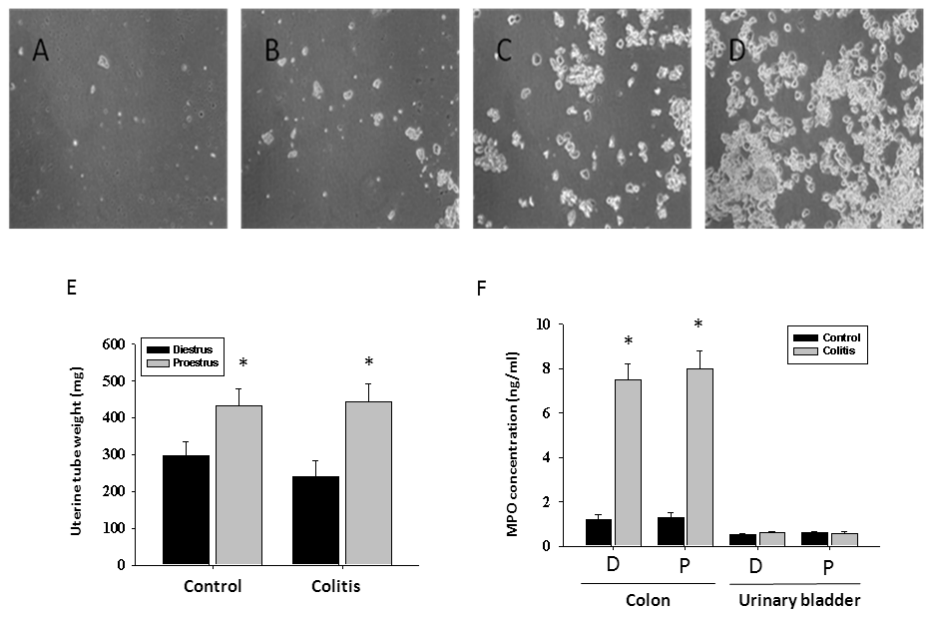
Evaluation of the stage of the estrous cycle in cycling female rats. Panels A–D show representative microphotographs of vaginal cytological samples taken from a female rat during diestrus 1 (metestrus, A), diestrus 2 (B), proestrus (C), and estrus (D). E, The uterine tube weight from rats in proestrus (N = 7) is significantly greater than in rats in diestrus (N = 6, p≤0.05) in both control and experimental groups of animals. F, Concentration of myeloperoxidase (MPO) enzyme is significantly elevated in the distal colon during colonic inflammation and unchanged in the urinary bladder of rats with active colitis. * - p≤0.05 to respective control group. D-diestrus, P-proestrus.

The phases of the estrous cycle were followed for 3 cycles in all female rats with an average duration of the cycle from 4 to 5 days. Once the exact pattern of the phases of the estrous cycle was established for each rat, the treatment with TNBS to induce colonic inflammation was done 5 days prior to the desired phase of the estrous cycle on the day of tissue harvesting. Animals had vaginal smears taken throughout the entire period of the study, and only tissues from animals in diestrus/metestrus (N = 6) and proestrus (N = 7) were harvested for neuropeptide analysis. Since metestrus only lasts for a short period of time (5–6 h) and the plasma estrogen concentration in metestrus did not differ from that in diestrus [28], data from these two groups of rats were pooled. Fig. 1 (A–D) represents the cytological evaluation of the cells in vaginal smears corresponding to the respective phases of the estrous cycle. The weight of the uterine horns was measured and used as the second index for determination of the estrous phase. Uterine horns from rats in proestrus were thicker, filled with fluid and weighed significantly more than ones from rats in diestrus (Fig. 1 E, p≤0.05 to diestrus). Induction of experimental colitis had no effect on the length of the phases of the estrous cycle nor on the weight of the uterine horns within the same phase of the cycle (Fig. 1 E).

### Myeloperoxidase Assay

The MPO assay is a validated biochemical method of grading inflammation in the tissue and measures the amount of MPO enzyme released by neutrophils at the site of inflammation [33]. The analysis of the enzyme concentration in the distal colon revealed a significant inflammatory reaction associated with 7-fold increase in MPO after TNBS treatment (Fig. 1F, p≤0.05 to control group). This increase in MPO was independent of the estrous cycle phase in rats with inflamed colon. The MPO values in the urinary bladder were not affected by TNBS instillation suggestive of the absence of detectable inflammation in the bladder (Fig. 1F).

### Ovarian Hormones Modulate CGRP Content in the Pelvic Organs and Sensory Ganglia

**Figure 2:**
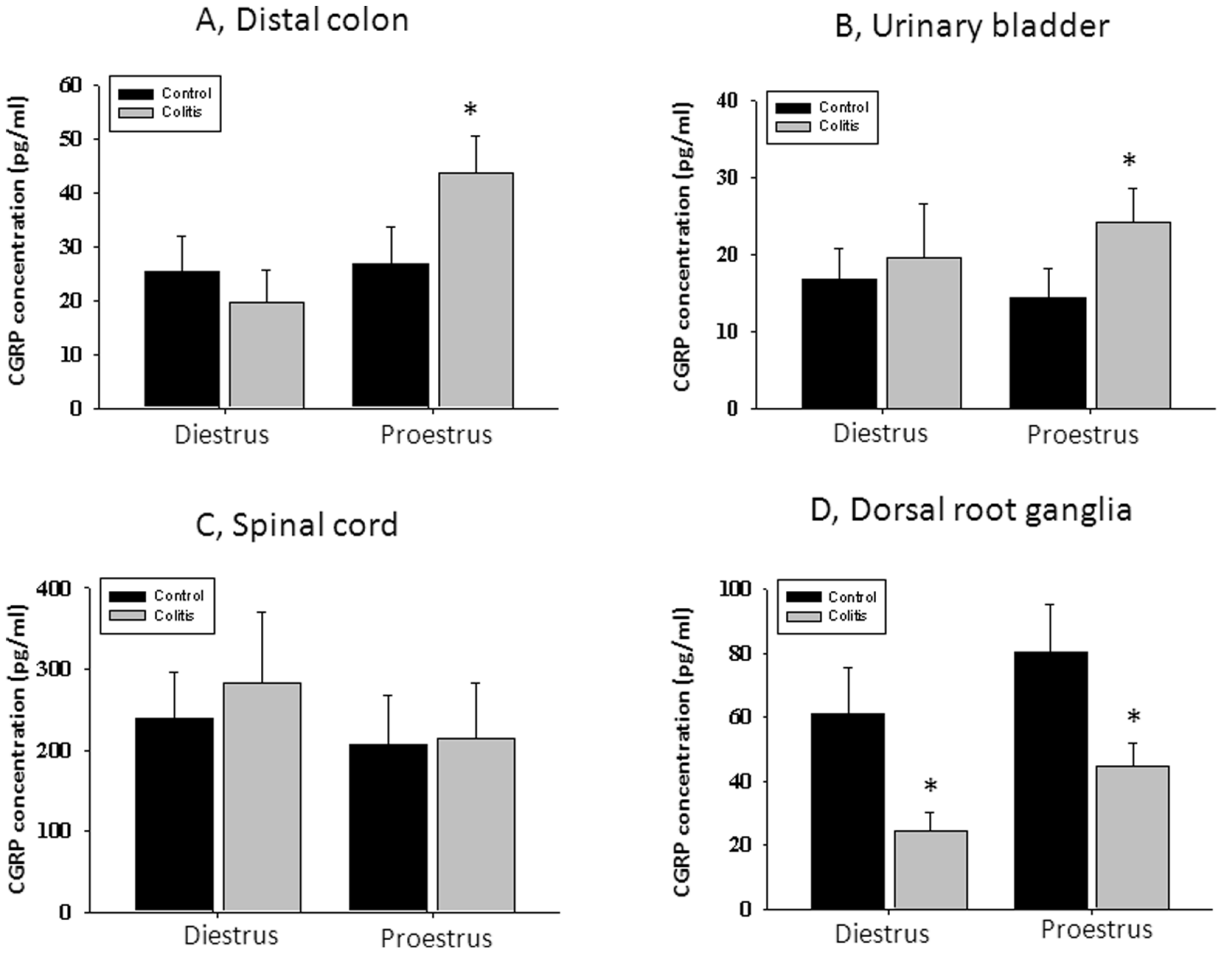
Estrous cycle dependent changes in CGRP content in the pelvic viscera and neural tissues after experimentally induced colitis. A, CGRP content in the distal colon during low (diestrus) and high (proestrus) estrogen. B, Concentration of CGRP in the urinary bladder during diestrus and proestrus phases. C, Neither phase of the estrous cycle nor colonic treatment significantly modulated CGRP content in the lumbosacral spinal cord. D, CGRP fluctuations within the estrous cycle in L6-S2 sensory ganglia innervating the pelvic organs. * - p≤0.05 to respective control group.

Comparison of CGRP level in the tested pelvic organs of control females during diestrus and proestrus did not reveal significant changes between these phases of the estrous cycle (Fig. 2 A and B). Protein concentration of CGRP in the distal colon (directly affected by inflammatory insult) was not different between control and inflamed groups during diestrus phase of the cycle (Fig. 2A). However, in rats with high estrogen at the time of testing (proestrus), experimental colitis caused a significant up-regulation of CGRP content by 63% (Fig. 2A, p≤0.05 to respective control). Transient colitis had similar effects on protein concentration of CGRP in the urinary bladder which was also up-regulated in rats during proestrus but not in diestrus when compared with control groups (Fig. 2B). Neither phase of the estrous cycle nor inflammatory insult had an effect on the CGRP content in the lumbosacral spinal cord (Fig. 2C). However, the concentration of CGRP was significantly diminished during both diestrus and proestrus in lumbosacral DRG after TNBS treatment (Fig. 2D) which may be associated with the release of this neuropeptide in the pelvic organs triggered by visceral inflammation.

### Visceral Cross-sensitization Up-regulates SP in the Pelvic Organs

**Figure 3:**
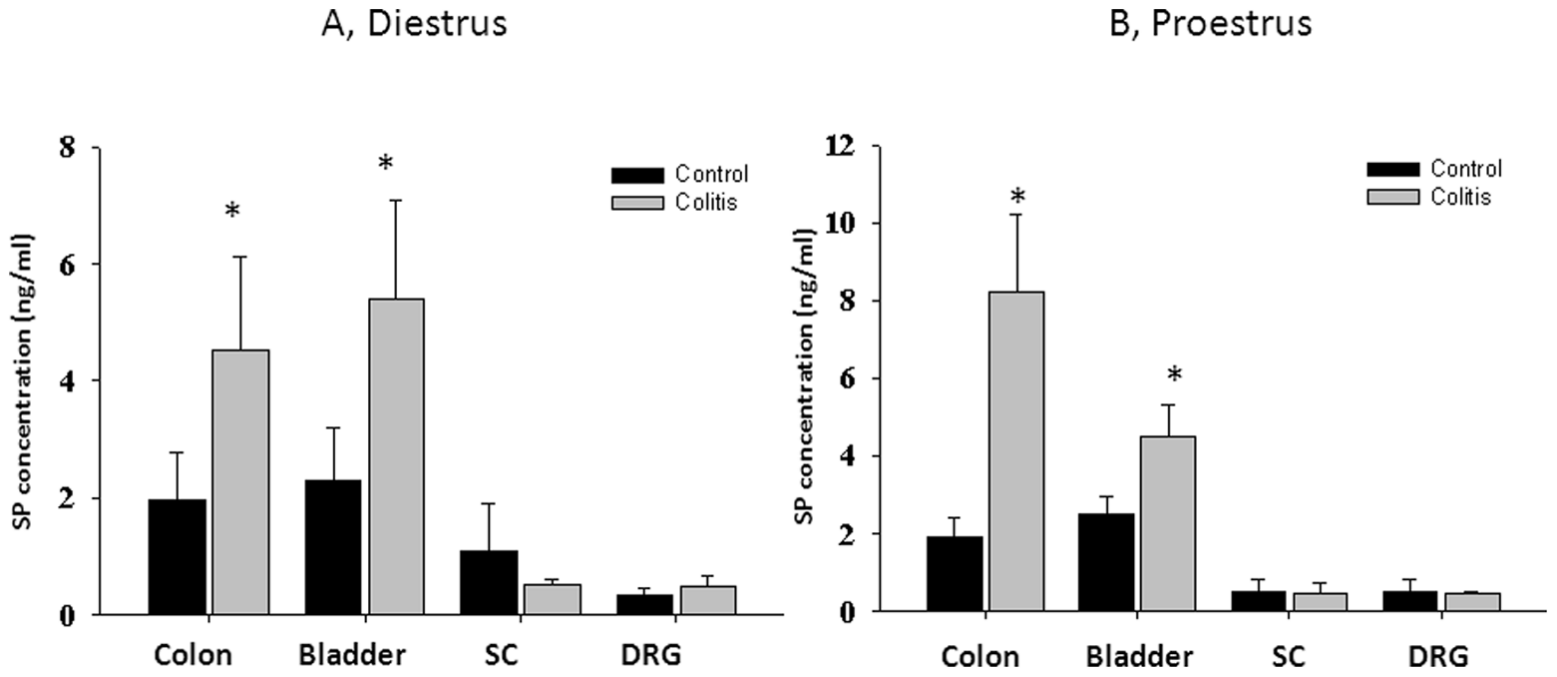
Visceral cross-sensitization up-regulates SP in the pelvic organs. A, Concentration of SP protein in the distal colon, urinary bladder and neural tissues in rats during diestrus. B, Substance P content during proestrus in control and TNBS-treated animals. SC- spinal cord, DRG – dorsal root ganglia. * - p≤0.05 to respective control group.

Under normal physiological conditions, concentration of SP was similar in the distal colon and urinary bladder during all phases of the estrous cycle. Development of inflammation in the colon caused a significant increase in the SP content by 2-fold in both organs (from 1.9±0.8 ng/ml to 4.4±2.1 ng/ml in the colon; from 2.3±0.9 ng/ml to 5.2±2.2 ng/ml in the bladder) during diestrus phase (Fig. 3 A, p≤0.05 to respective control). Similar changes were observed in the urinary bladder of rats in proestrus (Fig. 3B). Concentration of SP in the distal colon during proestrus was elevated 4-fold in comparison with untreated animals (Fig. 3 B, N = 7, p≤0.05 to control).

### BDNF Levels are Modulated by Ovarian Hormones during Active Colitis

**Figure 4:**
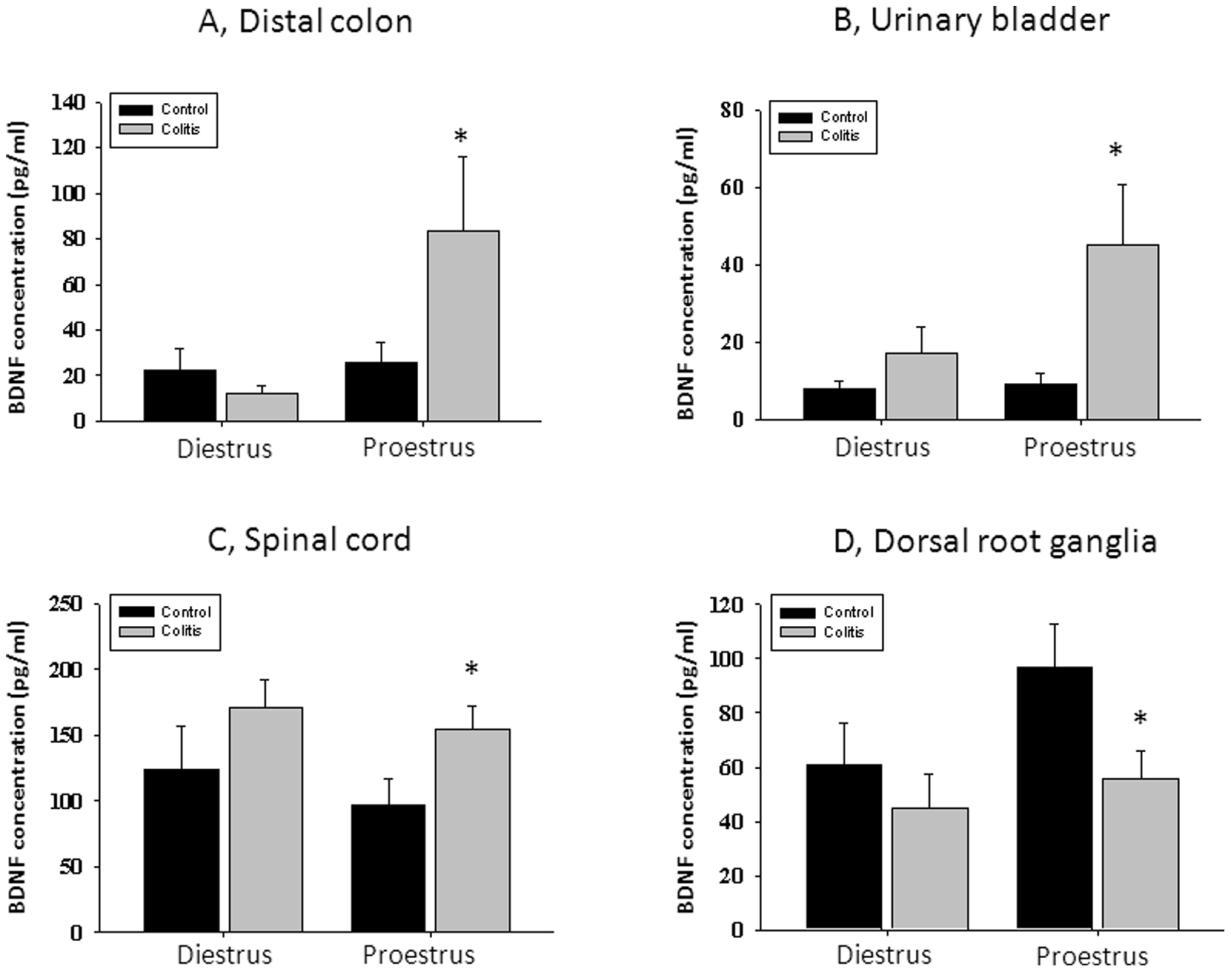
Protein levels of BDNF are modulated by ovarian hormones during active colitis. A, BDNF content in the distal colon during low (diestrus) and high (proestrus) estrogen. B, Concentration of BDNF in the urinary bladder during diestrus and proestrus phases. C, Experimentally induced colitis significantly up-regulated BDNF content in the lumbosacral spinal cord during proestrus phase. D, BDNF fluctuations within the estrous cycle in L6-S2 sensory ganglia innervating the pelvic organs. * - p≤0.05 to respective control group.

Protein concentration of BDNF in the distal colon was not different between control and inflamed rats during diestrus phase of the cycle (Fig. 4 A). However, in rats with high estrogen at the time of testing (proestrus), experimental colitis caused a significant up-regulation of BDNF colonic content from 26.1±8.5 pg/ml to 83.4±32.5 pg/ml (3.2-fold increase, Fig. 4A,N = 7, p≤0.05 to respective control). Experimental colitis had similar effects on the level of BDNF in the urinary bladder which was also up-regulated by 5-fold in rats in proestrus (from 8.4±3.9 pg/ml to 43.8±15.6 pg/ml, p≤0.05 to control) but not in diestrus when compared with respective control groups (Fig. 4B). Colonic inflammation also induced an increase in BDNF content in the lumbosacral spinal cord but the difference reached statistical significance only for proestrus phase (Fig. 4C, p≤0.05 to respective control). In addition, colonic inflammation triggered a diminution of BDNF content in sensory ganglia during proestrus phase but not during diestrus phase of the estrous cycle (Fig. 2D).

### Limited Effects of Experimental Colitis on Concentration of NGF during Colon-bladder Cross-talk

**Figure 5:**
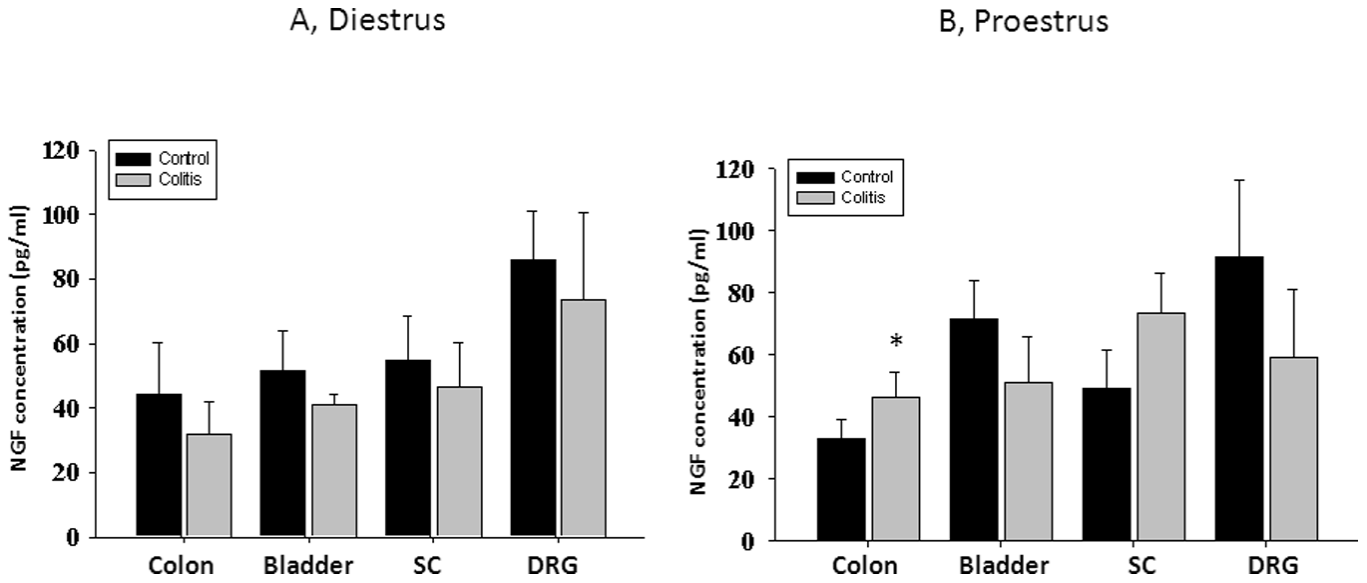
Limited effects of ovarian hormones on NGF concentration during colon-bladder cross-sensitization. A, Concentration of NGF protein in the distal colon, urinary bladder and neural tissues in rats during diestrus. B, NGF content during proestrus in control and TNBS-treated animals. SC- spinal cord, DRG – dorsal root ganglia. * - p≤0.05 to respective control group.

Concentration of NGF in isolated neural and pelvic tissue samples from female rats in diestrus and proestrus varied between the organs but has not been significantly affected by acute inflammation of the distal gut (Fig. 5). The only significant difference was observed between control and experimental groups in the distal colon in animals during proestrus (Fig. 5B). In this group, NGF content was elevated by 43% in comparison with untreated animals in the same phase of the estrous cycle (Fig. 5 B, N = 7, p≤0.05).

## Discussion

We have evaluated the estrous cycle dependent fluctuations of major regulatory neuropeptides involved in the development of neurogenic inflammation in the pelvis upon colon-bladder cross-sensitization. The results of our study provide evidence that ovarian hormones influence neuropeptide content in the pelvic organs of female animals and, therefore, may play a role in cyclic fluctuations of pain intensity and duration observed in women with BPS and other CPP disorders.

Steroid hormones show the capacity to modulate neurogenic inflammation and nociceptive signaling due to a widespread expression of estrogen receptors (ER) in the pelvic organs and associated neural pathways. Both estrogen receptors, ER-α and ER-β, have been detected within the bladder mucosa by immunohistochemical labeling, and ER-β is the predominant isoform expressed in the basal cell layer of the urothelium [34], [35]. Estrogen receptors are also expressed in the central and peripheral nervous system innervating the urogenital organs [36]. Previous studies established that estrogen deficiency in women, especially long-term, is associated with a wide range of urogenital complaints, including frequency, nocturia, incontinence, urinary tract infections and urgency (reviewed in [37]). Experiments in female rabbits determined that ovariectomy results in significantly decreased bladder contractile function followed by an increase in the ER density [38]. Inflammation of the lower urinary tract reduced the ER-β expression in the inflamed urinary bladder of rats and caused more severe inflammatory reaction in ovariectomized animals [39]. Additional studies determined that EB-β-deficient female but not male mice develop a bladder phenotype resembling human BPS [40].

Previous investigations established that estradiol can regulate the expression of preprotachykinin [23], CGRP [24], and NGF [25] receptors suggesting that estrogens likely influence a variety of peripheral functions via ER-mediated mechanisms [41]. In the present study, we observed estrous cycle dependent effects of experimental colitis on pro-inflammatory neuropeptides SP and CGRP which were significantly up-regulated in the urinary bladder during proestrus phase. Interestingly, concentration of SP was up-regulated in the pelvic organs of female rats during both low (diestrus) and high (proestrus) estrogen whereas CGRP levels were significantly higher only during proestrus phase. These results correlate with the data from our previous study performed on male rats which showed a significant up-regulation of SP but no significant changes in CGRP content in the pelvic organs at the same time point [9]. This data also matches the results published by Shaffer at al [30] who established no significant differences in CGRP and SP levels as a function of the estrous cycle in control rats but substantial enhancement of neuropeptide content in proestrus after bladder inflammation. It is highly likely that expression of CGRP is more estrogen dependent when compared to SP, however, additional studies are needed to understand the underlying mechanisms of such sensitivity.

Although it is unlikely that fluctuations in plasma estrogen concentrations alone stimulate pain and inflammation, estrogen may play a role in modulating the intensity of the response to neurogenic inflammation within the bladder. Recent work by Rudick *et al* evaluated the differences in pain responses between males and females using a pseudorabies virus-induced model of cystitis in mice [42]. They established that female mice showed significantly greater pelvic pain behavior in comparison with male mice upon development of neurogenic cystitis. However, ovariectomy and subsequent acute estrogen replacement had no effect on the magnitude of virus-induced neuropathic pain in female mice. It should be noted that the phases of the estrous cycle were not tracked in this study, nor were compared the pain responses between different phases of the estrous cycle in affected female mice. The presented results suggest that ovarian hormones play rather modulatory than key role in gender based pelvic pain in this model of neurogenic cystitis.

Previous studies determined that both ERα and ERβ mRNA levels were up-regulated during proestrus in comparison with metestrus in the sensory neurons of intact cycling female rats [43]. Low concentrations of 17β-estradiol, which acts via ER-α receptors, also increased survival of cultured DRG neurons deprived of NGF [44]. ER-α and ER-β stimulate different signaling pathways and, therefore, may differentially influence the extent and direction of neurotrophin expression within the estrous cycle [45]. There are a number of important factors that convey the neuropeptide effects including transport to and release from peripheral nerve terminals, rate of peptide metabolism, positive/negative feedback on synthesis and release [46]. Estrogen and the neurotrophins may influence each other’s actions by regulating receptor and ligand availability as well as by reciprocal regulation at the level of signal transduction or gene transcription. They can also stimulate the synthesis of proteins required for neuronal differentiation, survival and maintenance of function [47].

Estrous cycle dependent up-regulation of CGRP content in the urinary bladder and distal colon occurred in parallel with the similar changes observed for BDNF. Concentration of BDNF in the distal colon and urinary bladder was enhanced by peripheral inflammation during proestrus but not diestrus phase. Additionally, we detected an elevated concentration of BDNF in the spinal cord of females in proestrus. BDNF is synthesized in primary sensory neurons after peripheral inflammation where it facilitates intracellular signal transduction and gene expression at the dorsal horn of the spinal cord via anterograde transport [22], [48]. Modulation of visceral pain by BDNF was shown to be sex-dependent with facilitation of painful responses in female animals [49]. This effect may be associated with an increase in estrogen receptor binding sites in the lumbosacral spinal cord during proestrus in female rats [50]. The role of BDNF in bladder inflammation was evaluated by intrathecal injections of either a general Trk receptor antagonist or a BDNF scavenger [51]. Both treatments notably improved bladder function during acute cystitis. The study by Qiao et al [52] determined that the level of BDNF high affinity receptor TrkB is increased in bladder afferent neurons during colonic inflammation. The accumulation of TrkB in bladder sensory neurons may enhance the responsiveness of these neurons to BDNF, thus, leading to the changes in neuronal plasticity. In DRG culture, BDNF also increases the expression level of CGRP, an excitatory neurotransmitter that is up-regulated in bladder afferent neurons during colonic inflammation [52], suggesting a possible complimentary role of BDNF in modulating CGRP expression and associated bladder afferent excitability.

Expression of BDNF in sensory neurons innervating pelvic organs can be modulated by NGF. The role of NGF and respective TrkA receptors in regulating BDNF expression in the DRG was illustrated by previous studies showing that NGF treatment increases BDNF expression in the TrkA/CGRP peptidergic DRG neurons [53]. Several groups previously established that colonic inflammation increases the level of NGF and/or neural activity in the inflamed colon [14], [16], [54]. We also observed an up-regulation of NGF in the directly affected organ (colon) during proestrus but not diestrus phase. This may be explained by the lower level of the inflammatory reaction induced in our study as well as by indirect effects of transient colitis on bladder neuropeptides. Enhanced levels of CGRP and BDNF in the pelvic organs during proestrus were paralleled by a significant decrease of these neuropeptides in the sensory ganglia which corresponded with their peripheral release. Physiological experiments in female rats did not determine estrous influences on the micturition threshold in uninflamed bladder, but direct inflammation of the urinary bladder substantially lowered the micturition threshold in rats in proestrus [26]. Similarly, no significant differences in the magnitude of the visceromotor response to urinary bladder distension as a function of the phase of the estrous cycle were detected in adult female rats with non-inflamed bladders [55]. Experiments with extravasation of Evans Blue dye in rats revealed increased vascular permeability in the intact urinary bladder in proestrus after either colonic or uterine inflammation. However, these changes were insignificant in metestrus [27]. Multi- and single-unit recordings from gracile nucleus have demonstrated cycle-dependent changes of neuronal activity in response to tactile stimulation of the skin in the pelvic region and of the reproductive organs of female rats, but not in the the colon [56]. Altogether, these studies indicate that the level of regulatory neuropeptides in the lower urinary tract fluctuates during the estrous cycle and may affect urinary bladder sensitivity and function in female animals.

### Clinical Relevance and Conclusions

The prevalence of CPP and pain co-morbidities in women suggests involvement of ovarian hormones in modulation of pain intensity and transmission in the pelvic organs. Steroidal hormones have the capacity to modulate neurogenic inflammation in the lower urinary tract through interaction with a variety of mediators of pain and inflammation. The present findings illustrating correlation between fluctuations in regulatory neuropeptides and estrogen concentration during development of colon-bladder cross-sensitization provide evidence for endogenous modulation of bladder pain by ovarian hormones. Further studies are warranted to understand the molecular physiology of pelvic organ cross-sensitization and CPP in order to advance the generation of new pharmacological therapies for the treatment of pelvic pain disorders in female patients.

## References

[1] Butrick CW (2003) Interstitial cystitis and chronic pelvic pain: new insights in neuropathology, diagnosis, and treatment. Clin Obstet Gynecol 46: 811–823. 14595223 10.1097/00003081-200312000-00011

[2] MacGregor EA, Frith A, Ellis J, Aspinall L, Hackshaw A (2006) Incidence of migraine relative to menstrual cycle phases of rising and falling estrogen. Neurology 67: 2154–2158. 16971700 10.1212/01.wnl.0000233888.18228.19

[3] Zondervan KT, Yudkin PL, Vessey MP, Dawes MG, Barlow DH, et al (1999) Prevalence and incidence of chronic pelvic pain in primary care: evidence from a national general practice database. Br J Obstet Gynaecol 106: 1149–1155. 10549959 10.1111/j.1471-0528.1999.tb08140.x

[4] Parsons CL, Zupkas P, Parsons JK (2001) Intravesical potassium sensitivity in patients with interstitial cystitis and urethral syndrome. Urology 57: 428–432. 11248610 10.1016/s0090-4295(00)01110-9

[5] Aaron LA, Buchwald D (2001) A review of the evidence for overlap among unexplained clinical conditions. Ann Intern Med 134: 868–881. 11346323 10.7326/0003-4819-134-9_part_2-200105011-00011

[6] Alagiri M, Chottiner S, Ratner V, Slade D, Hanno PM (1997) Interstitial cystitis: unexplained associations with other chronic disease and pain syndromes. Urology 49: 52–57. 9146002 10.1016/s0090-4295(99)80332-x

[7] Sutcliffe S, Colditz GA, Pakpahan R, Bradley CS, Goodman MS, et al. (2013) Changes in symptoms during urologic chronic pelvic pain syndrome symptom flares: Findings from one site of the MAPP Research Network. Neurourol Urodyn doi: 10.1002/nau.22534. [Epub ahead of print]. 10.1002/nau.22534 PMC4032370 24273163

[8] Craft RM (2007) Modulation of pain by estrogens. Pain 132: S3–S12. 17951003 10.1016/j.pain.2007.09.028

[9] Pan XQ, Gonzalez JA, Chang S, Chacko S, Wein AJ, et al (2010) Experimental colitis triggers the release of substance P and calcitonin gene-related peptide in the urinary bladder via TRPV1 signaling pathways. Exp Neurol 225: 262–273. 20501335 10.1016/j.expneurol.2010.05.012 PMC2939259

[10] Lei Q, Pan XQ, Villamor AN, Asfaw TS, Chang S, et al (2013) Lack of transient receptor potential vanilloid 1 channel modulates the development of neurogenic bladder dysfunction induced by cross-sensitization in afferent pathways. J Neuroinflammation 10: 3. 23305398 10.1186/1742-2094-10-3 PMC3556132

[11] Bueno L, Fioramonti J (2002) Visceral perception: inflammatory and non-inflammatory mediators. Gut 51: i19–i23. 12077058 10.1136/gut.51.suppl_1.i19 PMC1867723

[12] Candenas L, Lecci A, Pinto FM, Patak E, Maggi CA, et al (2005) Tachykinins and tachykinin receptors: effects in the genitourinary tract. Life Sci 76: 835–862. 15589963 10.1016/j.lfs.2004.10.004

[13] Wesselmann U (2001) Neurogenic inflammation and chronic pelvic pain. World J Urol 19: 180–185. 11469605 10.1007/s003450100201

[14] di Mola FF, Friess H, Zhu ZW, Koliopanos A, Bley T, et al (2000) Nerve growth factor and Trk high affinity receptor (TrkA) gene expression in inflammatory bowel disease. Gut 46: 670–679. 10764711 10.1136/gut.46.5.670 PMC1727937

[15] Lowe EM, Anand P, Terenghi G, Williams-Chestnut RE, Sinicropi DV, et al (1997) Increased nerve growth factor levels in the urinary bladder of women with idiopathic sensory urgency and interstitial cystitis. Br J Urol 79: 572–577. 9126085 10.1046/j.1464-410x.1997.00097.x

[16] Qiao LY, Grider JR (2010) Colitis elicits differential changes in the expression levels of receptor tyrosine kinase TrkA and TrkB in colonic afferent neurons: a possible involvement of axonal transport. Pain 151: 117–127. 20638179 10.1016/j.pain.2010.06.029 PMC2939212

[17] Lin YT, Ro LS, Wang HL, Chen JC (2011) Up-regulation of dorsal root ganglia BDNF and trkB receptor in inflammatory pain: an in vivo and in vitro study. J Neuroinflammation 8: 126. 21958434 10.1186/1742-2094-8-126 PMC3203068

[18] Mannion RJ, Costigan M, Decosterd I, Amaya F, Ma QP, et al (1999) Neurotrophins: peripherally and centrally acting modulators of tactile stimulus-induced inflammatory pain hypersensitivity. Proc Natl Acad Sci U S A 96: 9385–9390. 10430952 10.1073/pnas.96.16.9385 PMC17792

[19] Xia CM, Gulick MA, Yu SJ, Grider JR, Murthy KS, et al (2012) Up-regulation of brain-derived neurotrophic factor in primary afferent pathway regulates colon-to-bladder cross-sensitization in rat. J Neuroinflammation 9: 30. 22335898 10.1186/1742-2094-9-30 PMC3298724

[20] Okragly AJ, Niles AL, Saban R, Schmidt D, Hoffman RL, et al (1999) Elevated tryptase, nerve growth factor, neurotrophin-3 and glial cell line-derived neurotrophic factor levels in the urine of interstitial cystitis and bladder cancer patients. J Urol 161: 438–441. 9915421

[21] Delafoy L, Gelot A, Ardid D, Eschalier A, Bertrand C, et al (2006) Interactive involvement of brain derived neurotrophic factor, nerve growth factor, and calcitonin gene related peptide in colonic hypersensitivity in the rat. Gut 55: 940–945. 16401692 10.1136/gut.2005.064063 PMC1856334

[22] Qiao LY, Gulick MA, Bowers J, Kuemmerle JF, Grider JR (2008) Differential changes in brain-derived neurotrophic factor and extracellular signal-regulated kinase in rat primary afferent pathways with colitis. Neurogastroenterol Motil 20: 928–938. 18373519 10.1111/j.1365-2982.2008.01119.x

[23] Liuzzi FJ, Bufton SM, Scoville SA (2001) Short-term estrogen replacement increases beta-preprotachykinin mRNA levels in uninjured dorsal root ganglion neurons, but not in axotomized neurons. Exp Neurol 170: 101–108. 11421587 10.1006/exnr.2001.7697

[24] Gangula PR, Lanlua P, Wimalawansa S, Supowit S, DiPette D, et al (2000) Regulation of calcitonin gene-related peptide expression in dorsal root ganglia of rats by female sex steroid hormones. Biol Reprod 62: 1033–1039. 10727274 10.1095/biolreprod62.4.1033

[25] Lanlua P, Decorti F, Gangula PR, Chung K, Taglialatela G, et al (2001) Female steroid hormones modulate receptors for nerve growth factor in rat dorsal root ganglia. Biol Reprod 64: 331–338. 11133691 10.1095/biolreprod64.1.331

[26] Johnson OL, Berkley KJ (2002) Estrous influences on micturition thresholds of the female rat before and after bladder inflammation. Am J Physiol Regul Integr Comp Physiol 282: R289–R294. 11742850 10.1152/ajpregu.2002.282.1.R289

[27] Winnard KP, Dmitrieva N, Berkley KJ (2006) Cross-organ interactions between reproductive, gastrointestinal, and urinary tracts: modulation by estrous stage and involvement of the hypogastric nerve. Am J Physiol Regul Integr Comp Physiol 291: R1592–R1601. 16946082 10.1152/ajpregu.00455.2006

[28] Becker JB, Arnold AP, Berkley KJ, Blaustein JD, Eckel LA, et al (2005) Strategies and methods for research on sex differences in brain and behavior. Endocrinology 146: 1650–1673. 15618360 10.1210/en.2004-1142

[29] Ji Y, Tang B, Traub RJ (2008) The visceromotor response to colorectal distention fluctuates with the estrous cycle in rats. Neuroscience 154: 1562–1567. 18550290 10.1016/j.neuroscience.2008.04.070 PMC2527482

[30] Shaffer AD, Ball CL, Robbins MT, Ness TJ, Randich A (2011) Effects of acute adult and early-in-life bladder inflammation on bladder neuropeptides in adult female rats. BMC Urol 11: 18. 21843346 10.1186/1471-2490-11-18 PMC3171712

[31] Bi R, Foy MR, Vouimba RM, Thompson RF, Baudry M (2001) Cyclic changes in estradiol regulate synaptic plasticity through the MAP kinase pathway. Proc Natl Acad Sci U S A 98: 13391–13395. 11687663 10.1073/pnas.241507698 PMC60881

[32] Malykhina AP, Qin C, Greenwood-van Meerveld B, Foreman RD, Lupu F, et al (2006) Hyperexcitability of convergent colon and bladder dorsal root ganglion neurons after colonic inflammation: mechanism for pelvic organ cross-talk. Neurogastroenterol Motil 18: 936–948. 16961697 10.1111/j.1365-2982.2006.00807.x

[33] Krawisz JE, Sharon P, Stenson WF (1984) Quantitative assay for acute intestinal inflammation based on myeloperoxidase activity. Assessment of inflammation in rat and hamster models. Gastroenterology 87: 1344–1350. 6092199

[34] Saunders PT, Maguire SM, Gaughan J, Millar MR (1997) Expression of oestrogen receptor beta (ER beta) in multiple rat tissues visualised by immunohistochemistry. J Endocrinol 154: R13–R16. 9379111 10.1677/joe.0.154r013

[35] Taylor AH, Al-Azzawi F (2000) Immunolocalisation of oestrogen receptor beta in human tissues. J Mol Endocrinol 24: 145–155. 10657006 10.1677/jme.0.0240145

[36] Papka RE, Storey-Workley M, Shughrue PJ, Merchenthaler I, Collins JJ, et al (2001) Estrogen receptor-alpha and beta- immunoreactivity and mRNA in neurons of sensory and autonomic ganglia and spinal cord. Cell Tissue Res 304: 193–214. 11396714 10.1007/s004410100363

[37] Hextall A, Cardozo L (2001) The role of estrogen supplementation in lower urinary tract dysfunction. Int Urogynecol J Pelvic Floor Dysfunct 12: 258–261. 11569655 10.1007/s001920170049

[38] Lin AD, Levin RM, Kogan BA, Whitbeck C, Leggett RE, et al (2006) Alteration of contractile and regulatory proteins in estrogen-induced hypertrophy of female rabbit bladder. Urology 68: 1139–1143. 17113912 10.1016/j.urology.2006.08.1094

[39] Terado M, Nomura M, Mineta K, Nishii H, Fujimoto N, et al (2005) Involvement of estrogen in the pathogenesis of cyclophosphamide-induced cystitis in rats. Endocrine 26: 55–63. 15805586 10.1385/ENDO:26:1:055

[40] Imamov O, Yakimchuk K, Morani A, Schwend T, Wada-Hiraike O, et al (2007) Estrogen receptor beta-deficient female mice develop a bladder phenotype resembling human interstitial cystitis. Proc Natl Acad Sci U S A 104: 9806–9809. 17522255 10.1073/pnas.0703410104 PMC1887607

[41] Purves-Tyson TD, Keast JR (2004) Rapid actions of estradiol on cyclic amp response-element binding protein phosphorylation in dorsal root ganglion neurons. Neuroscience 129: 629–637. 15541884 10.1016/j.neuroscience.2004.08.019

[42] Rudick CN, Pavlov VI, Chen MC, Klumpp DJ (2012) Gender specific pelvic pain severity in neurogenic cystitis. J Urol 187: 715–724. 22177208 10.1016/j.juro.2011.10.048

[43] Taleghany N, Sarajari S, DonCarlos LL, Gollapudi L, Oblinger MM (1999) Differential expression of estrogen receptor alpha and beta in rat dorsal root ganglion neurons. J Neurosci Res 57: 603–615. 10462685

[44] Patrone C, Andersson S, Korhonen L, Lindholm D (1999) Estrogen receptor-dependent regulation of sensory neuron survival in developing dorsal root ganglion. Proc Natl Acad Sci U S A 96: 10905–10910. 10485924 10.1073/pnas.96.19.10905 PMC17981

[45] Jezierski MK, Sohrabji F (2000) Region- and peptide-specific regulation of the neurotrophins by estrogen. Brain Res Mol Brain Res 85: 77–84. 11146109 10.1016/s0169-328x(00)00244-8

[46] Lundberg JM, Franco-Cereceda A, Alving K, Delay-Goyet P, Lou YP (1992) Release of calcitonin gene-related peptide from sensory neurons. Ann N Y Acad Sci 657: 187–193. 1637084 10.1111/j.1749-6632.1992.tb22767.x

[47] Toran-Allerand CD (1996) Mechanisms of estrogen action during neural development: mediation by interactions with the neurotrophins and their receptors? J Steroid Biochem Mol Biol 56: 169–178. 8603038 10.1016/0960-0760(95)00234-0

[48] Li WP, Xian C, Rush RA, Zhou XF (1999) Upregulation of brain-derived neurotrophic factor and neuropeptide Y in the dorsal ascending sensory pathway following sciatic nerve injury in rat. Neurosci Lett 260: 49–52. 10027697 10.1016/s0304-3940(98)00958-6

[49] Li F, Zhang JW, Wei R, Luo XG, Zhang JY, et al (2010) Sex-differential modulation of visceral pain by brain derived neurotrophic factor (BDNF) in rats. Neurosci Lett 478: 184–187. 20470867 10.1016/j.neulet.2010.05.013

[50] Williams SJ, Chung K, Om AS, Papka RE (1997) Cytosolic estrogen receptor concentrations in the lumbosacral spinal cord fluctuate during the estrous cycle. Life Sci 61: 2551–2559. 9416777 10.1016/s0024-3205(97)01009-6

[51] Frias B, Allen S, Dawbarn D, Charrua A, Cruz F, et al (2013) Brain-Derived Neurotrophic Factor, Acting at the Spinal Cord Level, Participates in Bladder Hyperactivity and Referred Pain during Chronic Bladder Inflammation. Neuroscience 234: 88–102. 23313710 10.1016/j.neuroscience.2012.12.044

[52] Qiao LY, Grider JR (2007) Up-regulation of calcitonin gene-related peptide and receptor tyrosine kinase TrkB in rat bladder afferent neurons following TNBS colitis. Exp Neurol 204: 667–679. 17303123 10.1016/j.expneurol.2006.12.024 PMC1906719

[53] Michael GJ, Averill S, Nitkunan A, Rattray M, Bennett DL, et al (1997) Nerve growth factor treatment increases brain-derived neurotrophic factor selectively in TrkA-expressing dorsal root ganglion cells and in their central terminations within the spinal cord. J Neurosci 17: 8476–8490. 9334420 10.1523/JNEUROSCI.17-21-08476.1997 PMC6573719

[54] Zhou XF, Rush RA (1996) Endogenous brain-derived neurotrophic factor is anterogradely transported in primary sensory neurons. Neuroscience 74: 945–951. 8895863 10.1016/0306-4522(96)00237-0

[55] Ball CL, Ness TJ, Randich A (2010) Opioid blockade and inflammation reveal estrous cycle effects on visceromotor reflexes evoked by bladder distention. J Urol 184: 1529–35. 20723927 10.1016/j.juro.2010.05.090

[56] Bradshaw HB, Berkley KJ (2000) Estrous changes in responses of rat gracile nucleus neurons to stimulation of skin and pelvic viscera. J Neurosci 20: 7722–7227. 11027234 10.1523/JNEUROSCI.20-20-07722.2000 PMC6772892

